# Socioeconomic pathways to inequalities in mental and functional health: a comparative study of three birth cohorts

**DOI:** 10.1186/s12889-020-10154-0

**Published:** 2021-01-19

**Authors:** Silvia Simone Klokgieters, Martijn Huisman, Marjolein Broese van Groenou, Almar Andreas Leonardus Kok

**Affiliations:** 1 Department of Epidemiology & Biostatistics, Amsterdam UMC – Location VU University Medical Center, De Boelelaan 1089a, 1081 HV Amsterdam, the Netherlands; 2 Department of Sociology, Faculty of Social Sciences, Vrije Universiteit, De Boelelaan 1105, 1081 HV Amsterdam, The Netherlands; 3 Department of Psychiatry, Amsterdam Public Health, Amsterdam UMC, Vrije Universiteit Amsterdam, Oldenaller 1, 1081 HJ Amsterdam, the Netherlands

**Keywords:** Socioeconomic inequalities, Structural equation modelling, Cohort differences, Daily functional limitations, Depressive symptoms

## Abstract

**Background:**

Although the educational expansion is often seen as a mechanism that might reduce health inequalities, socioeconomic inequalities in health (SEIH) have persisted or increased over the past decades. Theories suggest that this persistence could be due to a changing role of education as a ‘gatekeeper’ to access other socioeconomic resources such as occupation and income that are also associated with health outcomes. To test this, we examine whether the mediating role of occupation and income in the education–health relationship differs between three cohorts of 55–64 year old adults.

**Methods:**

We used cross-sectional data from three cohorts of 988, 1002, and 1023 adults born in 1928/37, 1938/47 and 1948/57 and observed in 1992/93, 2002/03, 2012/13 respectively, who participated in the Longitudinal Aging Study Amsterdam, the Netherlands. We used multigroup structural equation modelling to compare the strength of indirect effects of education via occupational skill level and income to functional limitations and depressive symptoms between cohorts.

**Results:**

Absolute educational inequalities in functional limitations increased for men and women in later cohorts, and in depressive symptoms only for men. Relative inequalities in functional limitations increased only for women and in depressive symptoms only for men. The indirect effect of education via income on both health outcomes was weaker in the most recent birth cohort compared to the earlier cohorts. In contrast, the indirect effect of education via occupation on functional limitations was stronger in the most recent cohort compared to the earlier cohorts. These differences were mainly due to a decreasing direct effect of education on income and an increasing direct effect of education on occupational skill level, rather than to changes in the direct effects of occupation and income on health.

**Conclusions:**

The role of education in determining inequalities in health appears to have changed across cohorts. While education became a less important determinant of income, it became a more important determinant of occupational level. This changing role of education in producing health inequalities should be considered in research and policy.

## Background

Socioeconomic inequalities in health (SEIH) have been remarkably resistant to policies and interventions in most industrialised societies. Recent time trend studies show stability or even an increase in absolute and/or relative socioeconomic inequalities in several health outcomes [1-4]. At the same time, significant changes in social stratification have been observed [1]. Throughout the twentieth century, access to education has improved substantially, resulting in an expansion of the number of higher educated individuals. Education is strongly related to other socioeconomic resources, such as higher skilled occupations and higher income, and both factors also have effects on health [2]. In fact, educational inequalities in health can be partly explained by the associations with income and occupation [3]. The fact that relatively many people achieved a high educational level in recent birth cohorts may have changed the way in which lower and higher educated individuals compete for jobs and gain access to higher wages [4]. This raises the question whether these changes have implications for health inequalities; specifically, whether the direct effects and indirect effects of education (via occupation and income) on health have changed over time. Therefore, this study examines whether the strength of indirect effects from education via occupational level and income on physical and mental health, have changed between three subsequent 10-year birth cohorts. We also examine to what extent potential changes in these indirect effects are due to changes in the direct effects of education on occupation and income and to changes in the direct effects of the socioeconomic indicators on health.

Fundamental cause theory (FCT) states that socioeconomic inequalities persist across time and place because social conditions are ‘fundamental causes’ of health and disease [5]. This means that the precise mechanisms and proximal risk factors that link socioeconomic inequalities to health may change over time, but those with a higher socioeconomic position consistently have better health outcomes because they have access to ‘flexible resources’ that promote health, such as money, knowledge, prestige and power [6].

Education is regarded as one of the most ‘fundamental’ among social causes of disease, because it is usually the first individual socioeconomic characteristic obtained in life, which subsequently provides access to most of the ‘flexible resources’ stipulated by FCT [7]. For example, higher educated individuals have access to less physically demanding and higher status jobs, with better financial resources, higher autonomy and more opportunities for further development [2]. Furthermore, the relationship between education and health is substantially mediated by occupation and income [3]. In addition, jobs with a higher occupational level typically provide more income [4]. As such, the relationship between education and income can also be assumed to be mediated through occupation. Therefore, education can be seen as a ‘gateway’ to further socioeconomic rewards (i.e. occupation and income) and – for a large part via these pathways – to better health [8]. Based on FCT, we expect that educational inequalities are persistent across time because a high level of education continues to provide access to flexible resources that ultimately improve health. However, several societal changes across the past decades suggest that the ways in which education operates as a gatekeeper to further socioeconomic resources and better health may have changed.

Exactly how they have changed remains uncertain and different theories seem to suggest different changes. Modernization theory argues that education grants access to human capital [9]. Individuals invest in schooling because education enhances their productivity and skills, which are rewarded by employers in the labour market [10]. It is assumed that each person with the same amount of skills is rewarded equally and that educational returns automatically increase when there is more demand than supply. Modernization theory thus states that educational attainment has become more important in modern economies as the demand for higher educated individuals has increased. Many jobs are more technologically advanced and industries demand more productivity and skills [11]. At least in western industrialized countries, the percentage of high skilled jobs has expanded over time, and these jobs are associated with higher income. If this theory is true, the effects of education on occupational skill level and income may have increased, leading to stronger indirect effects of education on health, via occupation and income.

In contrast, the positional model of education presumes that there has been an inflation of educational credentials that decreases the occupational [12] and economic returns [13] from education. The model argues that the employment structure has failed to keep up with the educational expansion [14]. Therefore, there are not enough high-skilled jobs available for the growing number of highly educated individuals. As a consequence, highly educated individuals are increasingly forced to compete for low skilled jobs. Moreover, as education is seen as a positional good, employers generally favour higher educated individuals over lower educated individuals, even if their specific skills for the job are identical [15]. Furthermore, because more high skilled jobs are available, the financial rewards offered for these jobs may decrease. Following this model, education can no longer guarantee high occupational and economic returns, and this development may neutralize some of the assumed advantage of higher educational qualifications. So, the positional model would suggest that the indirect effects of education on health via occupation and income are weaker in recent generations.

### Current study

We use repeated cross-sectional data from three independent birth cohorts (born 1928/37, 1938/47 and 1948/57), each observed 10 years apart. We argue that changes in the roles of explanatory factors in educational inequalities result from a culmination of cohort and period effects. We examine cohorts aged 55–64 years, an age-range which represents a moment in the life course where people are often still employed but at the same time start developing initial health problems. As such, socioeconomic inequalities in some respects are at their highest in this age group [16]. The birth years of the cohorts overlap with different stages of the educational expansion which started around the 1950s [17]. If we assume that adults finish their education by the age of 18 [3] then the youngest birth cohorts finished their education before the educational expansion started. The second and third birth cohorts finished their education during and after the educational expansion between 1956 and 65 and 1966–75 respectively. In addition, existing studies show that cohort differences in health inequalities are often larger among women than among men. This has been ascribed to developments such as emancipation, increasing female employment rates and role differences [18]. Therefore, we conducted the analyses separately for men and women.

## Methods

The Longitudinal Aging Study Amsterdam (LASA) is an ongoing longitudinal, multidisciplinary study, that focuses on physical, cognitive, social, and emotional functioning of Dutch older adults [19, 20]. Respondents were randomly selected from the population registers of 11 urban and rural municipalities in the west, northeast and south of the Netherlands. Trained interviewers visited respondents at home and data were collected by means of personal interviews that lasted approximately 2 hours. We selected the baseline measurement and two refresher measurements for inclusion in the study. The baseline measurement was collected in 1992 and included 3107 respondents aged 55–84 years old (cooperation rate was 62%). The first refresher cohort was collected in 2002–2003 and included 1002 adults aged between 55 and 64 years (cooperation rate was 62%). The third refresher cohort was collected in 2012–2013 and consisted of 1023 adults also aged between 55 and 64 years (cooperation rate was 63%). Given our focus on comparing independent groups of adults of the same age but observed at different moments in time (cross-sectional), we selected only those individuals aged between 55 and 64 years from the baseline measurement, which yielded 988 respondents. We, henceforward, refer to the cohorts by birth year which are 1928/37, 1938/47 and 1948/57 respectively. Included were 472 men and 516 women in the 1928/37-cohort, 475 men and 527 women in the 1938/47-cohort, and 496 men and 526 women in the 1948/57-cohort.

### Measures

#### Socioeconomic indicators

*Education* consisted of nine categories indicating the highest obtained qualification. We recoded the categories to the nominal number of years needed to obtain the qualification, ranging from 5 to 18 (5= *elementary not completed* to 18= *university education*).

*Occupation* consisted of five skill levels ranging from 1 to 5 (1=*elementary* to 5=*scientific*). Respondents were asked to give their current, last, and longest occupation. Occupations were coded using the Standard Classification of Occupation (SBC92) derived from the Statistics Netherlands (CBS) [21]. Highest achieved occupation was used. If a respondent indicated that he/she never had a job, respondents were categorized in a “Never paid job” category. Occupation was included in the models as continuous variable and never paid job was separately included as dummy variable.

*Income* of the household was categorized in 13 categories ranging from less than €454 to €2268 or more, at baseline. In order to make income comparable among all respondents (with or without partner), the midpoint amount of each income category is multiplied by 0.7 for respondents who indicated that both they themselves and their partner contribute to the monthly income. This correction makes all incomes equivalent to one-person household incomes. It is based on the ratio in Dutch state pensions for citizens living alone and living with others [22]. If income data was missing, it was imputed using data from subsequent measurement waves of LASA. For the 1928/37-cohort this resulted in 123 (1995–1996), 19 (1998–1999), and 9 (2001–2002) imputations, for the 1938/47-cohort in 43 (2005–2006), 11 (2008–2009), and 5 (2011–2012) imputations and for the 1948/57-cohort in 33 (2015–2016) imputations. All imputed income values were adjusted for inflation, which was on average 2.7, 1.9 and 1.4% per year in 1992–2001, 2002–2011, and 2012–2016, respectively. In descriptive tables, median values are shown. In multivariate analysis, income was expressed in hundreds of euros (range 3.17–24.39).

#### Health outcomes

*Functional limitations* was based on the extent to which respondents indicated that they were able to perform six daily activities: walking up and down a fifteen-step staircase without resting, getting dressed and undressed, sitting down and getting up from a chair, cutting their own toenails, walking 5 minutes outdoors without resting, and driving or using public transport. Response categories ranged from 0 to 4 (0=*No, I cannot* to 4=*Yes without difficulty*). Because the residuals were not normally distributed, all activities were dichotomized in 0 (= *none with difficulty*) and 1 (=*one or more activities with difficulty*).

*Depressive symptoms* used the 20-item Center for Epidemiologic Studies Depression Scale (CESD− 20; Radloff, 1977). Response categories included 0–3 (0=*rarely or never* to 3=*mostly or always*), resulting in a sum score ranging from 0 to 60. Because the residuals non-normally distributed sum scores the previously established clinically significant cut-off of 16 or more [23] was used for dichotomization.

### Procedure

For reasons of acquiring optimal statistical power, missing data on socioeconomic indicators was imputed using multiple imputation. We averaged values obtained from 20 imputed data sets in SPSS. The number of imputed values was two for education, 109 for income, and five for occupation. Comparison of results between models with and without imputed data revealed no differences. Respondents who had missing data on functional limitations (*n* = 4) and depressive symptoms (*n* = 17) were excluded from analysis using the outcome.

Our analytical procedure followed three steps. First, we examined descriptive statistics of education, occupation, income and health outcomes in each cohort. We tested differences in unadjusted means, percentages, and variances of these variables across cohorts, stratified for gender, which gave a preliminary indication of cohort changes.

Second, linear and logistic regression models were used to estimate the total effect of socioeconomic indicators on functional limitations and depressive symptoms. Models were adjusted for age. Next, based on our underlying theoretical model, constructed a model in Mplus version 7 [24]. The starting point was a model with a health outcome and education only. Subsequently, the complexity of the model was increased until we arrived at our final model that was saturated with all possible effects between education, occupation, income, and health. This process was performed for each health outcome separately. The indirect (i.e. mediation) effects were obtained through a bootstrapping procedure with 1000 draws [25], resulting in bootstrapped 95% confidence intervals Indirect effects depict the degree to which education influences health through other socioeconomic indicators of occupation and income. If the strength of the indirect effect increased across cohorts, this indicates that the explanatory role of the mediator has become more important across cohorts, and vice versa for weaker indirect effects.

Subsequently, through multigroup modelling we investigated whether the strength of indirect effects differed between cohorts by testing equality of indirect effects between the cohorts using a chi square difference test after imposing parameter constrains on the model. Because the full model included both dichotomous and continuous variables, we used a weighted least squares with mean adjustment estimator. This estimator uses the maximum number of observations available for each mediation in the model.

### Sensitivity analysis

Because the main analysis with raw education variables does not take into account that the proportion of higher educated has become higher in later cohorts, we estimated all models again using the Relative Index of Inequality of education (RII) [26], to see if this would affect our conclusions. The RII adjusts for changes in the population share of economic groups.

## Results

### Descriptive analysis

The average level of education, income and occupation increased across generations (Table 1). Men and women gained, on average, 1 year of education in each subsequent cohort. The percentage of men and women with a scientific occupation nearly doubled from the 1928/37-cohort to the 1948/57-cohort and the income level increased from the 1938/47-cohort to the 1948/57-cohort. Among women, fewer individuals never had a paid job (15%, 7%, and 9% respectively) whereas that percentage was very low in men in all birth cohorts (3%, 1%, and 4% respectively). The percentage of men with functional limitations increased over the three cohorts and the percentage of women with functional limitations increased from the 1928/37-cohort to the 1938/47-cohort and then decreased from the 1938/47-cohort to the 1948/57-cohort. The percentage of women with depressive symptoms was highest in the 1938/47-cohort, there were no cohort differences in depressive symptoms among the men.

**Table 1:** Characteristics of 55–64 year olds in three birth cohorts (1928–1937, 1938–1947, 1948–1957)

	Men					Women				
		1928/37-cohort	1938/47-cohort	1948/57-cohort	*p* Value		1928/37-cohort	1938/47-cohort	1948/57-cohort	*p* Value
	N	M /% (SD)	M /% (SD)	M /% (SD)	t/F test	N	M /% (SD)	M /% (SD)	M /% (SD)	t/F test
Age	1443	60 (3)	60 (3)	60 (3)		1570	60 (3)	60 (3)	60 (3)	
Functional limitations (%)										
Yes	358	19	26	30	< 0.001	455	18	36	33	< 0.001
Depressive symptoms (%)										
Yes	120	8	9	8	> 0.05	220	10	18	14	< 0.05
Education (in years)	1443	10 (3)	11 (4)	12 (4)	< 0.001	1568	9 (3)	10 (3)	11 (3)	< 0.001
Education (%)										
Elementary not completed	245	23	19	10	< 0.001	392	41	24	10	< 0.001
High	423	20	31	36		256	8	13	27	
Occupational level (%)										
Elementary	68	5	5	5	< 0.001	156	12	11	11	< 0.001
Low	287	22	21	19		513	48	41	20	
Medium	568	48	41	33		474	29	32	39	
High	350	17	27	30		215	8	13	24	
Scientific	133	8	7	14		52	3	2	6	
Never had a paid job (%)										
Yes	37	3	1	4	< 0.05	160	15	7	9	< 0.05
Income (€317- €2439)	1443	1247 (458)	1245 (355)	1669 (401)	< 0.001	1570	915 (379)	1047 (342)	1536 (398)	< 0.001

### Trends in SEIH: total effects

Educational differences in the prevalence of functional limitations increased in the later birth cohorts for men and women (Table 2). Educational inequalities for depressive symptoms similarly increased for men but decreased slightly for women in the 1948/57-cohort.

**Table 2:** Prevalence and rate difference of functional limitations and depressive symptoms per level of education across cohorts

Level of education	Men			Women		
	1928/37-cohort	1938/47-cohort	1948/57-cohort	1928/37-cohort	1938/47-cohort	1948/57-cohort
Functional limitations						
Education (in %)						
Elementary not completed	31	41	48	21	45	62
High	14	16	20	16	17	21
**Rate difference**	**17**	**25**	**28**	**5**	**28**	**41**
Depressive symptoms						
Education (in %)						
Elementary not completed	12	14	18	11	24	11
High	8	8	5	9	9	13
**Rate difference**	**4**	**6**	**13**	**2**	**15**	**-2**

**Fig. 1:**
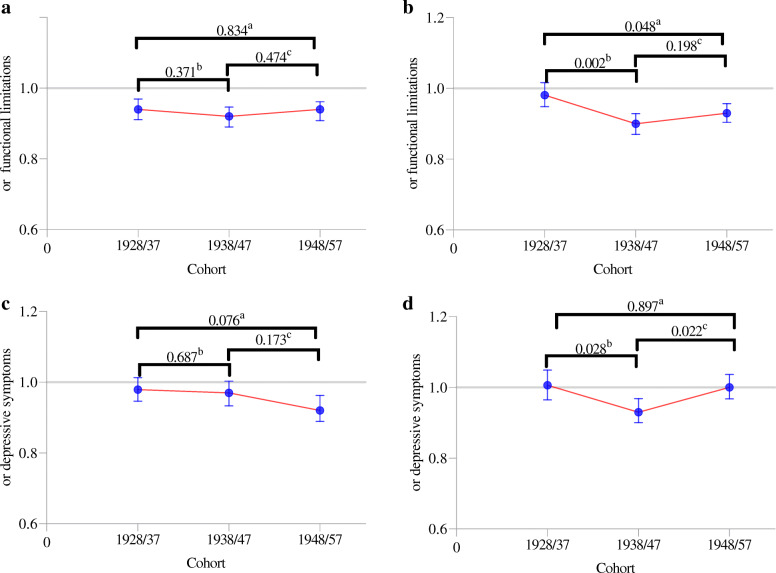
Age-standardized OR of health outcomes by education (range 5–18) and sex. **a**. Functional limitations, men. **b**. Functional limitations, women. **c**. Depressive symptoms, men. **d**. Depressive symptoms, women. Notes: ^a^ *P*-value of Wald difference test between 1928/37-cohort and 1948/57-cohort. ^b^ *P*-value of Wald difference test between 1928/37-cohort and 1938/47-cohort. ^c^ *P*-value of Wald difference test between 1938/47-cohort and 1948/57-cohort.

Relative educational inequalities in functional limitations went up for both men and women (Fig. 1a-d). However, statistical difference testing across cohorts only revealed differences between the 1928/37-cohort (OR = 0.98; 95% CI [0.95, 1.02]) and the 1938/47-cohort (OR = 0.90; 95% CI [0,87, 0,93], *p* = 0.002) and between the 1928/37-cohort and the 1948/57-cohort (OR = 0.93; 95% CI [0.90,0.96], *p* = 0.048) among women (Fig. 1b). Among women, relative educational inequalities in depressive symptoms were largest in the 1938/47-cohort (OR = 0.93; 95% CI [0.90, 0.97]) but returned in the 1948/57-cohort to a level comparable to the1928/37-cohort (Fig. 1d).

### Trends in indirect effects from education to health

**Fig. 2:**
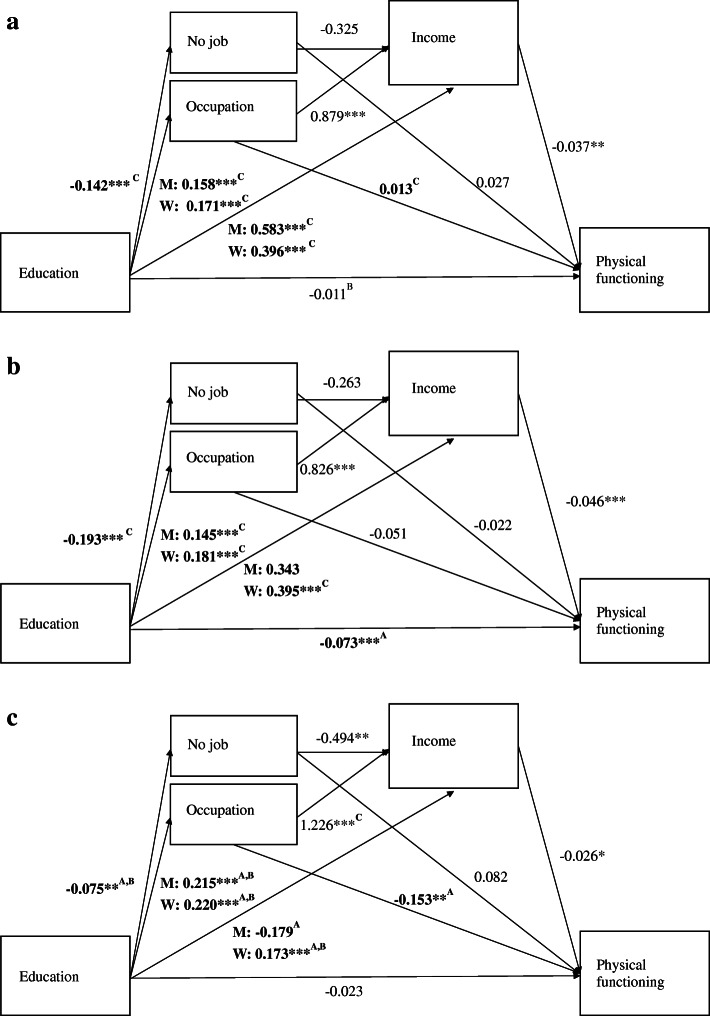
Causal relation between education and functional limitations across cohorts. a. Functional limitations, 1928/37-cohort. b. Functional limitations, 1938/47-cohort. c. Functional limitations, 1948/57-cohort. Notes. *p < .05, **p < .01. A = different from 1928/37-cohort, B =different from 1938/47-cohort, C = different from 1948/57-cohort. Comparison across cohorts were based on a Wald difference tests and was considered statistically significant if *p* < .01. All coefficients are controlled for age

**Fig. 3:**
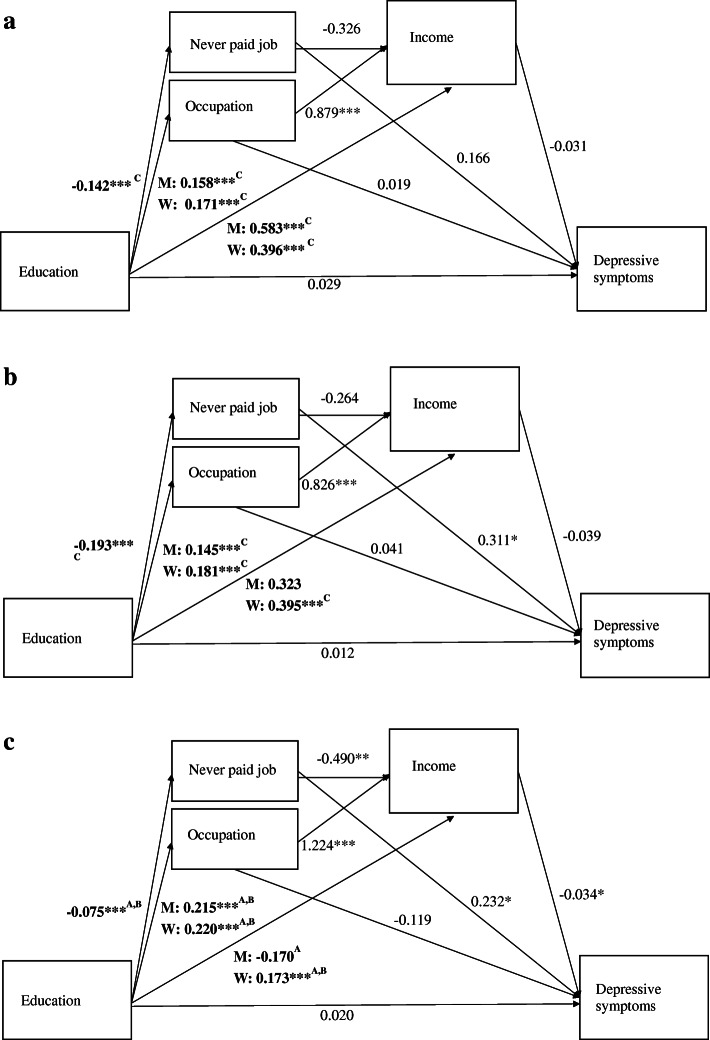
Causal pathways between education and depressive symptoms across cohorts. **a**. Depressive symptoms, 1928/37-cohort. **b**. Depressive symptoms, 1938/47-cohort. **c**. Depressive symptoms, 1948/57cohort. Notes. *p < .05, **p < .01, ***p < .001. A = different from 1928/37-cohort, B =different from 1938/47-cohort, C = different from 1948/57-cohort. Comparison across cohorts were based on a Wald difference tests and was considered statistically significant if *p* < .01. All coefficients are controlled for age

For each of the health outcomes and for each of the cohorts, three two-part indirect effects and two three-part indirect effects were estimated. Indirect effects are depicted in Table 3. In addition, in order to infer which direct effect induced a change in the indirect effect, Figs. 2 and 3 depict the unstandardized estimates of direct paths for each cohort. Single headed arrows indicate the assumed causal direction between variables and letters indicate whether or not an effect differed statistically significantly across cohorts. Two indirect effects were significantly different across cohorts. First, for both health outcomes, the indirect effect from education via income was lowest in the 1948/57-cohort. For functional limitations, this change occurred from the 1928/37-cohort (*b* = − 0.019, *p* < .01) to the 1948/57-cohort (*b* = − 0.001, *p* > .05) and from the 1938/47-cohort (*b* = − 0.014, *p* < .05) to the 1948/57-cohort (Table 3). For depressive symptoms this occurred from the 1938/47- cohort (*b* = − 0.011, (*p* > .10) to the 1948/57-cohort (*b* = − 0.001, *p* > .10). Second, the indirect effect of education via occupation on functional limitations was higher in the 1948/57-cohort (*b* = − 0.034, *p* < .01) than in the 1928/37-cohort (*b* = 0.002, *p* > .10).

**Table 3:** Indirect effects via socioeconomic indicators across cohorts

	1928/37-cohort	1938/47-cohort	1948/57-cohort
Indirect from education to functional limitations			
Via never paid job	−0.004	0.004	−0.006
Via occupational level	0.002^C^	−0.009	−0.034**^A^
Via income	−0.019**^C^	−0.014**^C^	− 0.001^A,B^
Via occupational level and income	−0.006*	− 0.006	−0.007*
Via income and never paid job	0.002	− 0.002	−0.001
Sum of indirect effect	−0.028	−0.027	− 0.048**
Indirect effect from education to Depressive symptoms			
Via never paid job	−0.024	−0.060†	− 0.017†
Via occupational level	0.003	0.007	−0.026
Via income	−0.015†	−0.011^C^	− 0.001^B^
Via occupational level and income	−0.005†	− 0.005	−0.009**
Via income and never paid job	−0.001	−0.002	− 0.001
Sum of indirect effect	−0.042*	− 0.072**	−0.055**

All coefficients are controlled for age

† *p* < .10. *p < .05. **p < .01

^A^ different from 1928/37-cohort. ^B^ different from 1938/47-cohort. ^C^ different from 1948/57-cohort

Exploring the sources of the changes in these indirect effects, depicted in Figs. 2 and 3, we found that the direct effect of education on income was lower in the 1948/57-cohort than in the 1928/37-cohort among men and lower in 1948/57-cohort than in 1928/37-cohort and in the 1938/47-cohort among women, for both functional limitations and depressive symptoms (Figs. 2 and 3). At the same time, the direct effect of income on functional limitations and the direct effect of income on depressive symptoms was not statistically significantly different across cohorts. This suggests that the decrease in the strength of the indirect effect of education on health via income occurred because the strength of the direct effect of education on income decreased.

For occupation, we found that the direct effect of education on occupational skill level was higher in the 1948/57-cohort compared to the 1928/37-cohort and the 1938/47-cohort (Figs. 2 and 3). Furthermore, the effect of occupation on functional limitations increased from the 1928/37-cohort to the to the 1948/57-cohort. This suggests that the increase in the indirect effect of education on functional limitations *through* occupation can be explained by an increase in both elements of this indirect effect, that is: the direct effect from education on occupation and from occupation on functional limitations both increased in more recent cohorts.

After controlling for mediation by occupation and income, the direct effect of education on the on the health outcomes fell below the threshold of statistical significance for all cohorts (Figs. 2 and 3). One exception was the effect of education on functional limitations in the 1938/47-cohort, which remained even after controlling for mediation (*b* = − 0.073, *p* < .001, Fig. 1b). This suggests that there is still an effect of education on functional limitations in the 1938/47-cohort while the effect of education on functional limitations and depressive symptoms is completely mediated by occupation and income in the two other cohorts.

### Sensitivity analysis

The analysis with the RII instead of education revealed similar results. The indirect effect from education via occupation increased across cohorts for functional limitations, from the1928/37-cohort (*b* = 0.034, *p* > .05) to the 1948/57-cohort (*b* = − 0.091, *p* > .05, difference: 95% CI [0.357, 0.939]) and from the 1938/47-cohort (*b* = − 0.618, *p* < .05) to the 1948/57-cohort, difference: 95% CI [0.235, 0.823]). The indirect effect from education via income decreased for functional limitations from the 1928/37-cohort (*b* = − 0.208, *p* < .01) to the 1948/57-cohort (*b* =− − 0.137, *p* < .01, difference: 95% CI [− 0.321, − 0.060]) and from the 1938/47-cohort (*b* = − 0.003, *p* < .05) to the 1948/57-cohort (difference: 95% CI [− 0.243, − 0.041]). The same was found for depressive symptoms, from the (1928/37-cohort (*b* = − 0.135, *p* > .05) to the 1948/57-cohort (*b* =− − 0.103, *p* > .05, difference: 95% CI [− 0.319, − 0.131]) and from the 1938/47-cohort (*b* = − 0.004, *p* > .05) to the 1948/57-cohort (difference: 95% CI [− 0.252, − 0.099]).

## Discussion

This study investigated differences between three birth cohorts of young-old adults born between 1928–1937, 1938–1947 and 1948–1957 in the mediating role of occupation and income in the education–health relationship. Results suggest that socioeconomic health inequalities have persisted but that the associations between the socioeconomic indicators producing them has changed between the cohorts.

Absolute and relative educational inequalities in functional limitations and depressive symptoms persisted or increased in later birth cohorts. However, changes in absolute inequalities were more pronounced than in relative inequalities. These findings are largely in line with prior studies [27–30]. Following FCT, we argue that education, in our study, indeed functions as a fundamental cause of disease as it remained stable across cohorts. We might infer that flexible resources, such as power, prestige and financial rewards, are, as such, continuously accessed through educational level.

We observed changes in two socioeconomic pathways to health. The indirect effect of education on functional limitations via occupation increased from the 1928/37-cohort to the 1948/57-cohort. The indirect effect of education on functional limitations via income decreased in later cohorts. In the introduction we argued that modernization theory and the positional model lead to opposing expectations; while modernization theory led us to expect that the effects of education on occupation and income increased, the positional model suggests that these effects would have remained stable or decreased. Our findings were partly consistent with both theories. Those with higher education became more likely to obtain a higher skilled job in more recent generations, supporting the modernization theory; it suggests that high-level occupational opportunities kept up with the educational expansion that occurred in the Netherlands around the 1950s [17]. As a consequence, education has become a more important determinant of people’s job skill level. However, the fact that education’s effect on income, after accounting for mediation, decreased across cohorts suggests that highly educated individuals only gain economic advantage over their lower educated peers when they also obtain a high level job. In earlier cohorts, the larger direct effect of education on income suggests that educational qualifications could, independently from occupational position, result in higher incomes, for example via on-the-job training or gradually increasing wages through the accumulation of experience in the same type of job. In more recent cohorts, it appears that such options may have become less accessible. The positional model suggests that there is credential inflation in the chances to obtain a job and in economic rewards [15]. While we found that the occupational structure seemingly kept up with the educational expansion, the economic returns from education in the absence of obtaining a higher skilled job decreased [13]. In addition, this decrease occurred more strongly for men than for women. As such, credential inflation may have occurred for economic returns from educational level but not through occupation.

In general, we found larger and more consistently increasing inequalities in functional limitations compared to depressive symptoms. This underscores the importance of examining multiple types of health outcomes in research on socioeconomic inequalities. One explanation for the differences may lie in differences in risk factors for these health outcomes. It has been shown that functional limitations are more strongly affected by health behaviours and depressive symptoms more strongly by factors such as stress exposure and social support [31]. Furthermore inequalities in health behaviours such as smoking [32] and alcohol use [33] increased in the past decades, whereas this is less so for psychosocial factors [34].

### Limitations

A few limitations of our design need to be mentioned. First, our study used data from three cohorts that were born only 10 years apart. It is possible that long-term consequences of the changing sociohistorical context have not been fully observed. Second, we used occupational skill-level as occupational indicator. However, this indicator captures only part of occupational status and neglects that there are many ways in which the labour market has changed throughout the twentieth century. For example, labour market segmentation theory posits issues such as increasing job insecurity, training and career opportunities, payment and job contents [35]. In addition, because we had no information available about how many household members contributed to the household income, we were not able to calculate household-per capital income. Therefore, it might be possible that we overestimated the income of respondents with more members than just the partner in the household. Third, we investigated cohort differences in repeated cross-sectional data. For convenience we referred to the three groups as ‘cohorts’, but it is possible that period, cohort and age effects all contribute to the effects that we found [36], although age effects may be minimal because the groups of 55–64 year old adults have already been robustly sorted into their socioeconomic categories due to a lifetime of advantage or disadvantage. Furthermore, it is likely that the societal developments that provide the background to our analysis also represent a mixture of cohort and period effects to which the individuals in our study were exposed throughout their lives, and it may – even if it would be analytically possible – not be theoretically or practically valuable to attempt to interpret these in isolation Fourth, we had no prior information available about the physical or mental health of our respondents. Therefore we were not able to account for potential health selection effects. For education this effect is probably limited [37]. However, for occupation and income, selection effects might play a role.

## Conclusions

We found that the magnitude of relative SEIH persisted and even increased in recent generations of 55–64 year old men and women. While the role of education as gatekeeper in health inequalities remains, its pathway through socioeconomic resources changed across cohorts. The pathway in which education determines health through income has partly been replaced by a pathway in which education determines health through occupational skill level. Apparently, it has become more difficult for lower educated individuals to obtain a higher skilled job, which increasingly and negatively affects their health in recent generations. An important takeaway for policy and intervention is to strengthen the occupational position of lower educated individuals through offering more opportunities for on-the-job training.

## Data Availability

Access to data from the Longitudinal Aging Study Amsterdam can be requested by submitting a LASA analysis proposal form for evaluation. The LASA evaluation committee provides access to the data on the condition that the goals of the data request are in keeping with the overarching aims of LASA that its participants have provided consent for. The LASA analysis proposal template includes the option to request data for replication purposes. The template of the analysis proposal form can be obtained at www.lasa-vu.nl, or by sending a request to the LASA secretariat, f.kursun@amsterdamumc.nl. Analysis proposals can be submitted to the LASA secretariat.

## Abbreviations

SEIH: Socioeconomic Inequalities in Health
FCT: Fundamental Cause Theory
LASA: Longitudinal Aging Study Amsterdam
RII: Relative Index of Inequality
